# Cationic Mechanosensitive Channels Mediate Trabecular Meshwork Responses to Cyclic Mechanical Stretch

**DOI:** 10.3389/fphar.2022.881286

**Published:** 2022-07-19

**Authors:** Susu Chen, Wenyan Wang, Qilong Cao, Shen Wu, Ningli Wang, Lixia Ji, Wei Zhu

**Affiliations:** ^1^ School of Pharmacy, Qingdao University, Qingdao, China; ^2^ Department of Clinical Pharmacy, The Second Hospital of Traditional Chinese Medicine of Huangdao District, Qingdao, China; ^3^ Qingdao Haier Biotech Co.,Ltd., Qingdao, China; ^4^ Beijing Institute of Ophthalmology, Beijing Tongren Hospital Eye Center, Capital Medical University, Beijing, China; ^5^ Beijing Advanced Innovation Center for Big Data-Based Precision Medicine, Beihang University and Capital Medical University, Beijing, China

**Keywords:** trabecular meshwork, cationic mechanosensitive channels, mechanical stretching, proteomics analysis, IOP homeostasis

## Abstract

The trabecular meshwork (TM) is responsible for intraocular pressure (IOP) homeostasis in the eye. The tissue senses IOP fluctuations and dynamically adapts to the mechanical changes to either increase or decrease aqueous humor outflow. Cationic mechanosensitive channels (CMCs) have been reported to play critical roles in mediating the TM responses to mechanical forces. However, how CMCs influence TM cellular function affect aqueous humor drainage is still elusive. In this study, human TM (HTM) cells were collected from a Chinese donor and subjected to cyclically equiaxial stretching with an amplitude of 20% at 1 Hz GsMTx4, a non-selective inhibitor for CMCs, was added to investigate the proteomic changes induced by CMCs in response to mechanical stretch of HTM. Gene ontology enrichment analysis demonstrated that inhibition of CMCs significantly influenced several biochemical pathways, including store-operated calcium channel activity, microtubule cytoskeleton polarity, toll-like receptor signaling pathway, and neuron cell fate specification. Through heatmap analysis, we grouped 148 differentially expressed proteins (DEPs) into 21 clusters and focused on four specific patterns associated with Ca^2+^ homeostasis, autophagy, cell cycle, and cell fate. Our results indicated that they might be the critical downstream signals of CMCs adapting to mechanical forces and mediating AH outflow.

## Introduction

The trabecular meshwork (TM), a small and complex tissue in the eye, maintains intraocular pressure (IOP) homeostasis through dynamic regulation of aqueous humor drainage (Goel et al., 2010; Acott et al., 2014; Buffault et al., 2020). Appropriate adaption of the TM to IOP fluctuations, including periodic ocular pulsation and other perturbations triggered by blinking, squeezing, rubbing, side-looking, and other activities, is extremely important for IOP homeostasis (Turner et al., 2019). Mechanical forces acting upon the TM have been reported to cause changes in autophagy (Hirt and Liton, 2017a), nitric oxide signaling (Stamer et al., 2011; Chandrawati et al., 2017), caveolin-1 signaling (Thorleifsson et al., 2010), and mechanosensitive ion channel activity (Tran et al., 2014; Yarishkin et al., 2019). Inappropriate adaption of the TM to the mechanical changes could potentially lead to increased resistance to aqueous humor drainage and elevated intraocular pressure (IOP), which is a strong risk factor for glaucoma (Acott et al., 2014).

Cationic mechanosensitive channels (CMCs), including transient receptor potential cation channel subfamily V member 4 (TRPV4) (Ryskamp et al., 2016), TWIK-related potassium channel-1 (TREK-1) (Carreon et al., 2017), and Piezo (Yarishkin et al., 2021; Zhu et al., 2021; Morozumi et al., 2022), have the potential to modulate calcium homeostasis, cell cytoskeleton organization, extracellular matrix (ECM) composition, and PGF2alpha secretion, and could therefore influence the responses of TM cells. In accordance with Yarishkin et al., we also found that GsMTx4, a non-selective inhibitor of CMCs, reduces the TM’s steady-state facility of AH outflow (Yarishkin et al., 2021; Zhu et al., 2021). However, CMC responses are very rapid and it is still not well understood how these channels act to maintain appropriate TM function in response to stretch.

Several groups have examined TM cell responses to biomechanical stretch using microarray and RNA sequencing technology. Youngblood et al. reported that mechanical stretch in the TM induces mRNA changes mainly associated with steroid biosynthesis, glycerolipid metabolism, and ECM-receptor interaction (Youngblood et al., 2020). Vittal et al. revealed that genes related to ECM modification, cytoskeletal regulation, and stress responses are notably induced in the TM by stretching (Vittal et al., 2005). However, these studies did not focus on the role of CMC signaling in TM cell stretching and furthermore did not investigate proteomic changes, which may differ from those observed at the transcriptional level.

To this end, we first investigated the proteomic changes in the TM in response to mechanical stretching through liquid chromatography with tandem mass spectrometry. We then explored CMC downstream signaling in response to stretch by blocking their function with GsMTx4.

## Materials and Methods

### Human Trabecular Meshwork (TM) Cell Isolation and Culture

As previously described (Zhu et al., 2016; Yu et al., 2019; Wang et al., 2021), human TM cells of one Chinese donor (Age 54; male without any ophthalmic diseases) obtained from Beijing Tongren Hospital (Beijing, China) were isolated, seeded onto the gelatin-coated (Sigma-Aldrich, St. Louis, MO) plates (Thermo Fisher Scientific, Waltham, MA), and maintained in human-complete medium comprised of medium 199E (Gibco, Grand Island, New York), 20% fetal bovine serum (FBS; Gibco), 90 μg/ml porcine heparin (Sigma-Aldrich), 20 U/ml endothelial growth factor supplement (Sigma-Aldrich) and 1.7 mM l-glutamine (Sigma-Aldrich). TM cells were cultured at 37°C with 5% CO_2_ and characterized at passages two to three by verifying TM biomarkers expression (Ding et al., 2014; Wang et al., 2021) and dexamethasone-inducible myocilin secretion (Yu et al., 2019; Wang et al., 2021). The use of human TM cells was approved by the ethics committee of Qingdao University and Beijing Tongren Hospital, following the use guidelines of the Medical College of Qingdao University and Beijing Tongren Hospital.

### Cyclic Mechanical Stretch

Human TM cells (HTM) at passage three and HTM5, an immortalized cell type derived from the human TM (Pang et al., 1994), were seeded onto collagen I (Sigma-Aldrich)-coated culture plates (Flexcell International Corporation, Burlington, NC) and starved in low serum medium comprised of α-MEM (Gibco) and 1% FBS (Gibco) for 24 h. When HTM reached 80% confluency, equiaxial mechanical stretch with an amplitude of 20% at 1 Hz was cyclically applied by FlexcellFX-5000TM Tension System (Flexcell International Corporation, Burlington, NC) for 3 h. Since CMCs are sensitive to GsMTx4 in a concentration-dependent manner, GsMTx4 (20 μM, Abcam, Cambridge, MA) was added during the stretch (Zhu et al., 2021). PBS (Gibco) was used as the vehicle control. Thus three groups of cells were collected for the proteomic analysis: control without stretch (Treatment one; T1), cyclic stretch (Treatment two; T2), and cyclic stretch with GsMTx4 treatment (Treatment three; T3).

### Protein Extraction and Quantification Analysis

Proteins were extracted with RIPA lysis buffer (Thermo) and quantified with BCA Protein Assay Reagent Kit (Thermo). 25 μg proteins were denatured in NuPAGE-LDS sample buffer (Invitrogen, Carlsbad, CA, United States ) at 95°C for 5 min and separated on 8–17% sodium dodecyl sulfate (SDS)-acrylamide gel by electrophoresis (Stacking gel: 80 V for 40 min; Separating gel: 120 V for 120 min).

### Proteomic Analysis

• *Reducing the Protein and Blocking Cysteine*—100 μg protein was dissolved in 100 μL triethylammonium bicarbonate (PierceTM TEAB 100 mM) and reacted with 5 μL Tris (2-carboxyethyl) phosphine (PierceTM TCEP 200 mM) at 55°C for 1 h and 5 μL PierceTM iodoacetamide (375 mM) at room temperature for 30 min. For protein precipitation, pre-chilled (-20°C) acetone (Thermo) was subsequently added. The pellet was collected through centrifugation (8,000× *g*, 10 min, 4°C).
• *Protein Digestion*—100 μg acetone-precipitated pellet was resuspended in 100 μL PierceTM TEAB (100 mM) and digested with trypsin (2.5 μg PierceTM trypsin for 100 μg protein) at 37°C overnight.
• *Peptide Labeling*—Tandem Mass Tag (TMT) Label Reagent was equilibrated with PierceTM anhydrous acetonitrile and reacted with the digested peptide at room temperature for 1 h. Then 8 μL 5% PierceTM hydroxylamine was added to quench the reaction.
• *C18 column separation*—The labeled peptides were dried using a vacuum centrifuge, dissolved in Solution A containing 2% PierceTM acetonitrile (vol/vol in water; pH 10.0), and flowed through the Pierce™ C18 column. The elution solutions were made of different combinations of Solution A and B (90% PierceTM acetonitrile; vol/vol in water; pH 10.0) and listed in SupplementaryTable S1. 20 peptide fractionations (elutions from 24 to 63 min) were collected for liquid chromatography with tandem mass spectrometry (LC-MS/MS) analysis.
• *LC-MS/MS analysis*—Samples dissolved in solution C (0.1% PierceTM formic acid; vol/vol in water) were separated by using solution D (99.9% PierceTM acetonitrile; vol/vol in PierceTM formic acid) at a flow rate of 300 nL/min for 65 min (Supplementary Table S2). LC-MS/MS was carried out using Q Exactive mass spectrometer (m/z 350-1,600; Thermo). The experiments were performed in Beijing CapitalBio Technology Co., Ltd. (Beijing, China).

### Western Blotting (WB)

40 μg protein was boiled, loaded on a 5% sodium dodecyl sulfate (SDS)-acrylamide stacking gel, separated on a 10% SDS-acrylamide gel by electrophoresis, and eventually transferred to the polyvinyl difluoride membrane (PVDF; GE Healthcare Life Sciences China, Beijing, China). After blocking in Tris-buffered Saline-Tween-20 containing 5% non-fat milk powder, incubation with diluted primary antibodies (Supplementary Table S3) followed by the corresponding secondary antibody conjugated with horseradish peroxidase (HRP; Abcam), immunoreactive bands were visualized using the enhanced chemiluminescence detection kit (Thermo) and a ChemiDoc XRS + imaging system (Bio-Rad). Band intensity was quantified using Image Lab software (Bio-Rad) and normalized to beta-Actin (Abways Technology, ab0035, Shanghai, China). Each experiment contained four technical replicates.

### Bioinformatics and Statistical Analyses

Data analysis was performed using Proteome discoverer software (version 1.4; Thermo). Proteins among different samples with fold change ≥1.50 or ≤0.66 and *p* values <0.05 were considered as differentially expressed proteins (DEPs) and applied for Gene Ontology (GO) enrichment analysis based on the PANTHER database, KEGG pathway enrichment analysis, and heatmap analysis. One-way ANOVA was applied to evaluate expression changes between groups.

## Results

### Preparation of Samples for LC-MS/MS

**FIGURE 1:**
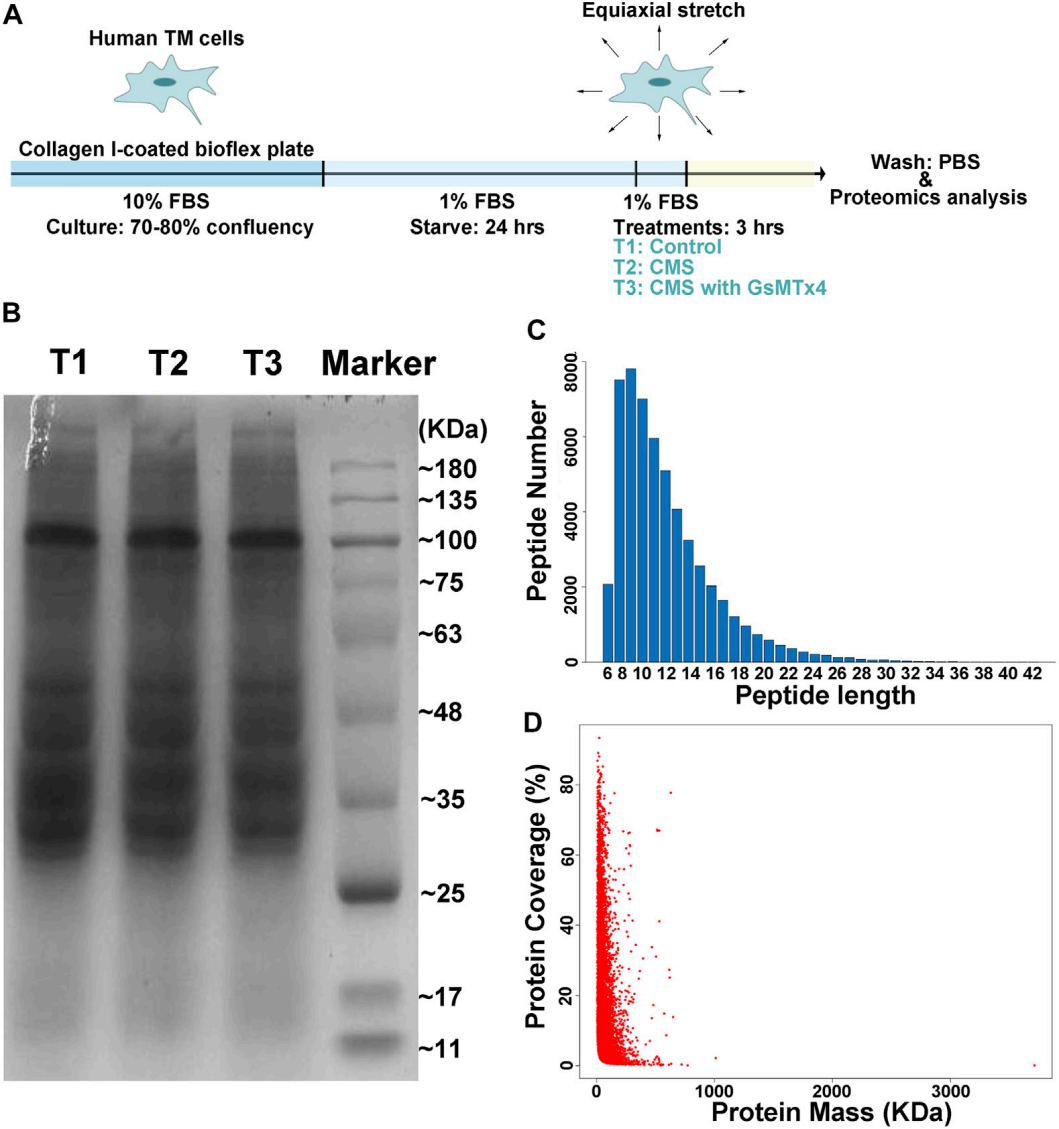
Schematic illustration of the experimental design. **(A)**. Schematic diagram of cell preparation, cyclic mechanical stretching, and sample collection. HTM cells are seeded onto the collagen I-coated plate, starved for 24 h, stretched cyclically with an amplitude of 20% at 1 Hz for 3 h, and collected for LC-MS/MS analysis, while GsMTx4 (20 μM) is applied to inhibit the function of CMCs. PBS is used as the vehicle control. T1: Control; T2: Cyclic mechanical stretching (CMS); T3: CMS with GsMTx4. In LC-MS/MS, the quality of our samples **(B)**, peptides lengths **(C)**, and protein coverage **(D)** are shown. Peptides with lengths from 6 to 51 amino acid residues cover over 80% of a protein.

As we previously demonstrated (Zhu et al., 2021), intracameral perfusion with GsMTx4, an inhibitor of cationic mechanosensitive channels (CMCs), leads to disturbances of conventional AH outflow. We predicted that a close relationship exists between CMCs and the TM’s biological responses to mechanical stretch. Therefore, proteomics analysis was performed to characterize this relationship by using HTM cells subjected to cyclic mechanical stretch with an amplitude of 20% at a frequency of 1 Hz for 3 h, a condition mirroring acute sustained elevation of IOP. GsMTx4 was applied at a concentration of 20 µM to inhibit CMC function (Figure 1A). We first determined the quality of our samples through SDS-acrylamide gel electrophoresis, peptide length, and protein coverage analyses (Figures 1B–D). Peptides with lengths from 6 to 51 amino acid residues covered over 80% sequence of a protein, indicating that it is successful and sufficient for the subsequential LC-MS/MS analysis.

### A Critical Role of CMCs in the TM in Response to Cyclic Stretch

**FIGURE 2:**
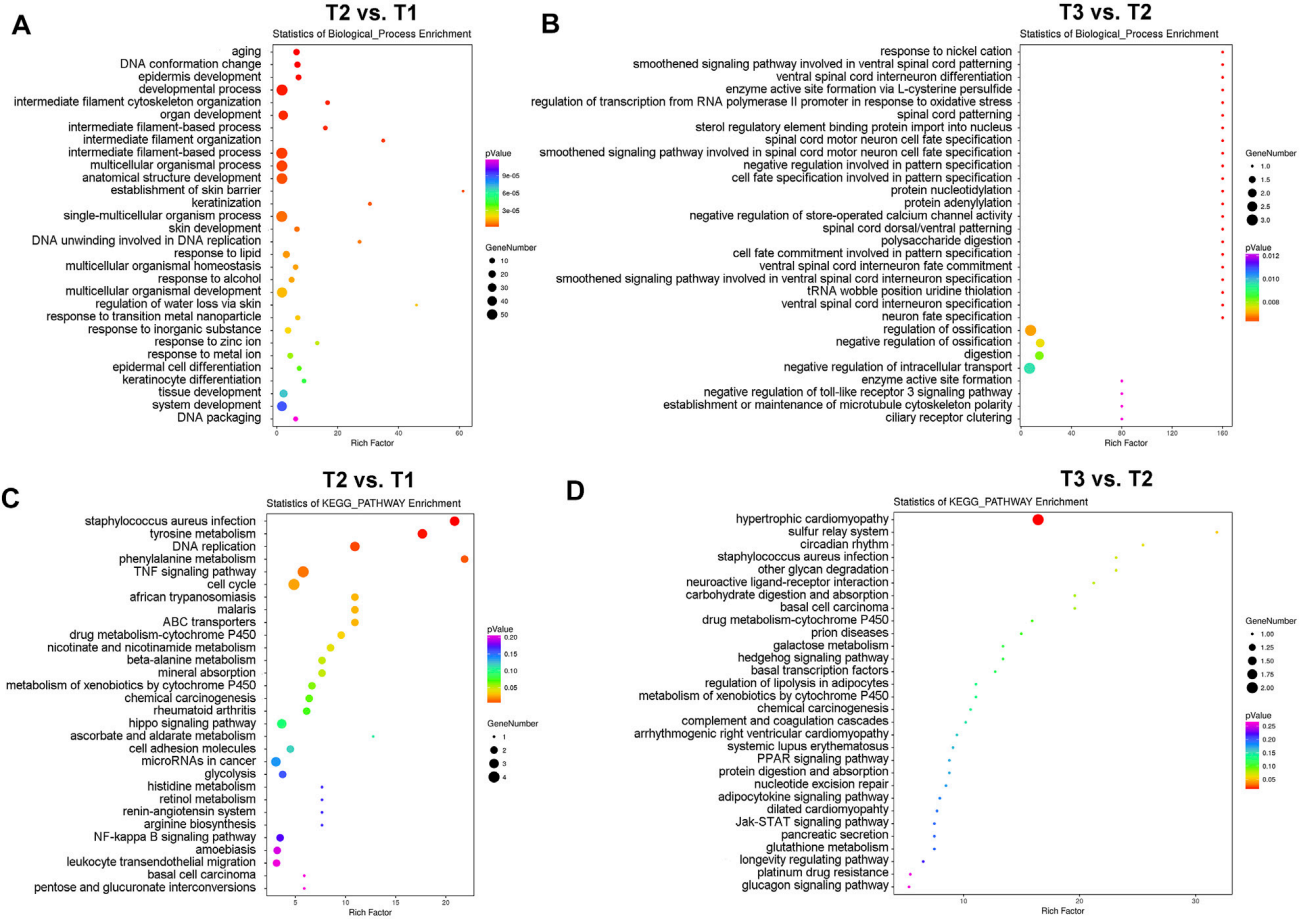
GO and KEGG pathway enrichment analyses of DEPs. DEPs with fold change ≥1.50 or ≤0.66 and *p* values <0.05 (T2 vs. T1: 26 up-regulated DEPs and 83 down-regulated DEPs; T3 vs. T2: 35 up-regulated DEPs and four down-regulated DEPs) are selected for GO **(A,B)** and KEGG pathway enrichment analyses **(C,D)**. Cyclic stretch evokes significant changes in the extracellular matrix organization, cytoskeleton remodeling, multicellular organismal homeostasis, metal ion response, aging, DNA conformation, keratinization, cell differentiation, cell cycle, and cell fate, which are inhibited by GsMTx4.

We next identified those differentially expressed proteins (DEPs) with fold change ≥1.50 or ≤0.66 and *p* values <0.05 (T2 vs. T1: 26 up-regulated DEPs and 83 down-regulated DEPs; T3 vs. T2: 35 up-regulated DEPs and four down-regulated DEPs) for subsequent bioinformatics analyses and pathway enrichment analyses. As shown in Figure 2A, cyclic stretch induces a significant up-regulation of proteins involved in extracellular matrix organization, cytoskeleton remodeling, multicellular organismal homeostasis, and metal ion response. These findings are similar to those reported previously (Youngblood et al., 2020). In addition, changes in proteins related to aging, DNA conformation, keratinization, cell differentiation, and tissue development were also found in the TM in response to cyclic stretch. Importantly, GsMTx4 treatment significantly altered expression changes (Figure 2B) and comparison of T1 and T2 detected expression changes of proteins related to store-operated calcium channel activity, microtubule cytoskeleton polarity, and toll-like receptor three signaling pathway. Intriguingly, neuron cell fate specification was another profound change in the TM after GsMTx4 treatment (Figure 2B).

In addition to biological process enrichment analysis, we performed KEGG pathway enrichment analysis to explore how CMCs function in the TM during mechanical stretch (Figures 2C, D). Expression of proteins involved in tyrosine metabolism, DNA replication, TNF signaling, cell cycle, and cytochrome P450 pathways were significantly changed after mechanical stretch, and GsMTx4 incubation led to significant changes in hypertrophic cardiomyopathy, sulfur relay system, and circadian rhythm.

### Downstream Signals Associated With CMCs in Response to Cyclic Stretch

**FIGURE 3:**
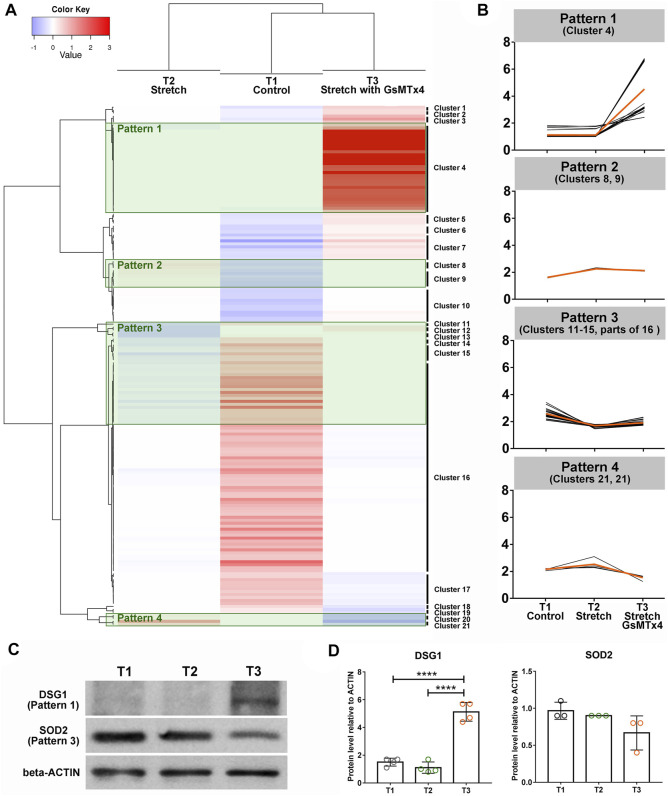
Heatmap analysis using DEPs. 148 DEPs (109 DEPs in T2 vs. T1 and 39 DEPs in T3 vs. T2) are grouped into 21 clusters **(A)**. Four specific patterns indicating the positive roles of CMCs in this process are shown **(B)**. The orange line indicates the average of DEPs’ expression changes in each group, while the black line denotes the expression of each DEP. **(C)**. Western blot analysis of DSG1 as a representative of Pattern 1, SOD2 as a representative of Pattern 3 (top) and beta-Actin (bottom) in T1, T2, and T3. **(D)**. Quantification of band intensities using Image Lab software (Bio-Rad). ****p < 0.0001 by One-way ANOVA.

To select the vital downstream signals of CMCs involved in HTM adapting to mechanical stretching, we subsequently grouped 148 DEPs (109 DEPs in T2 vs. T1 and 39 DEPs in T3 vs. T2) into 21 clusters (Figure 3A) and focused on four specific patterns (Figure 3B). We were particularly interested in DEPs that did not display any significant changes in response to cyclic stretching but showed a significantly increased expression after GsMTx4 treatment (Pattern 1). In Pattern 2, 3, and four mechanical stretching evoked a significant change in HTM, but GsMTx4 inhibited these stretch-induced changes (Figure 3B). In total 30 DEPs in Pattern 1, nine DEPs in Pattern 2, 34 DEPs in Pattern 3, and four DEPs in Pattern four were listed in Supplementary Tables S4–7 as downstream candidates of CMCs signaling in response to cyclic mechanical stretch. Those DEPs (Table 1) that are particularly likely to maintain TM function in response to mechanical stretching include two-pore calcium channel (TPCN1), desmoglein-1 preproprotein (DSG1), glutathione S-transferase (GSTT2), chromodomain-helicase-DNA-binding protein (CHD6), transcription factor jun-B (JUNB), connective tissue growth factor (CCN2), superoxide dismutase (SOD2), cytoskeleton protein vimentin (VIM), zinc finger protein (ZFN618), ubiquitin (HACE1), AMP-activated protein kinase (PRKAB1), as well as the ECM component collagen (COL8A1). We then verified expression changes of DSG1 and SOD2 as representative proteins for Patterns 1 and 3, respectively, by Western blot analysis. As shown in Figures 3C, D, DSG1 and SOD2 exhibited similar expression patterns as observed by LC-MS/MS (Figures 3A,B).

**TABLE 1:** Downstream candidates of CMCs in HTM adapting to mechanical stretching.

Pattern	ProbeSetId	Gene_Symbol	Gene_ID	Description	T1	T2	T3
Pattern 1	NP_001933.2	DSG1	1828	desmoglein-1 preproprotein [Homo sapiens]	1.01	1.01	6.69
Pattern 1	NP_001338275.1	TPCN1	53373	two pore calcium channel protein 1 isoform 3 [Homo sapiens]	1.01	1.01	6.68
Pattern 1	NP_001289599.1	GSTT2	2953	glutathione S-transferase theta-2 isoform b [Homo sapiens]	1.02	1.01	6.63
Pattern 1	NP_115597.3	CHD6	84181	chromodomain-helicase-DNA-binding protein 6 [Homo sapiens]	1.01	1.01	6.73
Pattern 2	NP_002220.1	JUNB	3726	transcription factor jun-B [Homo sapiens]	1.64	2.26	2.10
Pattern 2	NP_001892.1	CCN2	1490	connective tissue growth factor precursor [Homo sapiens]	1.59	2.34	2.09
Pattern 2	NP_001198.2	BTF3	689	transcription factor BTF3 isoform B [Homo sapiens]	1.66	2.26	2.08
Pattern 2	NP_001155046.1	MAFF	23764	transcription factor MafF isoform b [Homo sapiens]	1.66	2.22	2.12
Pattern 3	NP_001502.1	CXCL1	2919	growth-regulated alpha protein precursor [Homo sapiens]	2.21	1.69	2.16
Pattern 3	NP_588615.2	ZNF618	114991	zinc finger protein 618 isoform 1 [Homo sapiens]	2.38	1.75	1.97
Pattern 3	NP_001309749.1	SOD2	6648	superoxide dismutase [Mn], mitochondrial isoform E [Homo sapiens]	2.48	1.73	1.88
Pattern 3	NP_003371.2	VIM	7431	vimentin [Homo sapiens]	2.39	1.79	1.92
Pattern 3	NP_444513.1	DCD	117159	dermcidin isoform 1 preproprotein [Homo sapiens]	2.39	1.76	1.93
Pattern 3	NP_001264074.1	ZBTB10	65986	zinc finger and BTB domain-containing protein 10 isoform c [Homo sapiens]	2.47	1.80	1.86
Pattern 3	NP_057613.4	ATP8A2	51761	phospholipid-transporting ATPase IB isoform 1 [Homo sapiens]	2.46	1.79	1.88
Pattern 4	NP_065084.2	COL8A1	1295	collagen alpha-1(VIII) chain precursor [Homo sapiens]	2.21	2.32	1.60
Pattern 4	NP_001337489.1	HACE1	57531	E3 ubiquitin-protein ligase HACE1 isoform i [Homo sapiens]	2.21	2.28	1.63
Pattern 4	NP_002657.3	PLIN1	5346	perilipin-1 [Homo sapiens]	2.14	3.09	1.26
Pattern 4	NP_006244.2	PRKAB1	5564	5'-AMP-activated protein kinase subunit beta-1 [Homo sapiens]	2.05	2.41	1.65

## Discussion

Proper adaption of the TM to cyclic mechanical stretch and other perturbations in the eye is required for maintaining AH outflow and IOP homeostasis (Ramos et al., 2009; Turner et al., 2019). Recent investigations have found that cyclic mechanical stretch at an amplitude of 10–15%, a condition mimicking physiological forces, evokes many transcriptional changes associated with ECM turnover (Shearer and Crosson, 2002; Youngblood et al., 2020), cytoskeleton remodeling, autophagy (Shim et al., 2021a), calcium ion sequestration (Ryskamp et al., 2016; Uchida et al., 2021), cell-cycle regulation (Wang et al., 2013), and sterol and lipid metabolism (Uchida et al., 2021). Our study confirms many of these changes at a translational level (109 DEPs of T2 vs. T1). Most of the DEPs identified are involved in aging, intermediate filament cytoskeleton organization, lipid response, and zinc and metal ions responses. Examples include hyccin (FAM), Cell Division Cycle (CDC), connective tissue growth factor (CCN), zinc finger protein (ZNF), phospholipid-transporting ATPase (ATP), and growth-regulated alpha protein (CXCL). Furthermore, in our hands cyclic mechanical stretch also causes several changes in related to rhythmic processes, cell differentiation, and developmental processes (Figure 2), providing us a new insight into the molecular effects of mechanical forces.

CMCs have been identified to play a crucial role in fast signaling during mechanotransduction in many systems (Bowman et al., 2007), including TRPV4-modulated calcium TM cell cytoskeleton (Ryskamp et al., 2016) or TREK-1-induced alternation of TM extracellular matrix composition (Carreon et al., 2017). To investigate the role of CMCs in the TM as an adaptation to cyclic mechanical stretch, GsMTx4 is an attractive tool due its highly specific inhibition of mechanosensitive channels (Gnanasambandam et al., 2017; Suchyna, 2017). Notably, GsMTx4-induced proteomic changes are not only caused by the depolarization of some types of CMCs, such as Piezo- and TRP- channels, but are also influenced by the inhibition of several mechanoenzymes (Khairallah et al., 2012; Storch et al., 2012; Gnanasambandam et al., 2017; Suchyna, 2017). In addition, some CMCs with two-pore (2P) domains have been reported to be potentiated by GsMTx4 (Gnanasambandam et al., 2017). LC-MS/MS results showed that several GsMTx4-induced DEPs during mechanical stretching are involved in store-operated calcium channel activity and microtubule cytoskeleton polarity (Figure 2). These findings hold great significance for calcium signal and cytoskeleton remodeling as CMC-related signals in response to mechanical stretch. Moreover, we also found that the toll-like receptor three signaling pathway is another CMC-mediated signalling pathway. In accordance with our findings, cyclic mechanical stretch has been reported to activate MTOR/AKT1/SMAD2/3 on primary cilia of TM cells and thus mediate autophagy (Shim et al., 2021a). These data suggest that CMCs might act as an upstream signal of the MTOR/AKT1/SMAD2/3 pathway and autophagy. Finally, we found that several DEPs (T3 vs. T2) are involved in neuron cell fate specification, which might be a new mechanism of the TM to adapt to mechanical forces.

In addition to the pathway enrichment analysis, this study also aimed to discover key downstream DEPs of CMCs in response to cyclic mechanical stretch. In Pattern 1, complex I intermediate-associated protein 30 mitochondrial precursor (NDUFAF1) with a 6.48-fold increase (T3 vs. T2) might be one CMC-mediated downstream signal with functions in TM cell survival and IOP homeostasis. Data obtained in mice have indicated that mitochondrial abnormalities could be an early driver for the development of glaucoma (Williams et al., 2017). In Pattern 1 we also found changes in TPCN1 (6.61-fold increase T3 vs. T2) and DSG1 (6.62-fold increase T3 vs. T2), which have a significant capacity in modulating calcium homeostasis. Jablonsik et al. have demonstrated that single nucleotide polymorphisms of Cacna2d1, the subunit of alpha2/delta-1 in voltage-dependent calcium channel, are associated with intracellular Ca^2+^ concentration, cell contractility, cytoskeleton, and stiffness of the TM, which eventually lead to the pathogenesis of primary open-angle glaucoma (Chintalapudi et al., 2017). These findings provided significant insight into TPCN1 and DSG1 as critical downstream effectors of CMCs mediated adaptation to mechanical forces and maintenance of AH outflow. In addition, we found significant changes in the expression (T3 vs. T2) of CHD6 (6.69-fold increase; Pattern 1), JUNB (0.93-fold decrease; Pattern 2), and ZNF618 (1.13-fold increase; Pattern 3), all molecules that are associated with autophagy. As reported, the activation of autophagy is essential for mechanotransduction in the TM (Shim et al., 2021b). They might also be necessary for nitric oxide release (Hirt and Liton, 2017b), which is extremely critical in controlling outflow resistance and IOP homeostasis (Reina-Torres et al., 2021). We also identified some DEPs involved in cell cycle and cell fate specification, such as CCN2 (0.89-fold decrease; Pattern 2), ZNF618 (1.13-fold increase; Pattern 3), VIM (1.07-fold increase; Pattern 3), and PRKAB1 (0.69-fold decrease; Pattern 4). Their specific role in CMC mediated TM responses to stretch remains unclear and will be the topic of further investigation.

In summary, this study confirmed the previous transcriptomics findings of stretch-induced changes in the TM at a translational level. More importantly, we show that CMC downstream signaling influences the adaptive responses of the TM to mechanical changes. Notably, calcium signaling, cytoskeleton remodeling, autophagy, cell cycle, and cell fate are pathways involved in this CMC-mediated adaption.

## Data Availability

The datasets presented in this study can be found in online repositories. The names of the repository/repositories and accession number(s) can be found below: http://proteomecentral.proteomexchange.org/cgi/GetDataset, PXD032288.
