# Supplementary material for: Cationic Mechanosensitive Channels Mediate Trabecular Meshwork Responses to Cyclic Mechanical Stretch
Source: Front Pharmacol. 2022 Jul 19;13:881286. doi: 10.3389/fphar.2022.881286 (PMC9343793; doi:10.3389/fphar.2022.881286)
Supplement: Supplementary file 1 [file Table1.docx]

Table S1. Elution conditons for collecting 20 peptide fractionations

| Time (min) | Flow rate (mL/min) | Solution A | Solution B |
| --- | --- | --- | --- |
| 0 | 0.4 | 100% | 0% |
| 3 | 0.4 | 100% | 0% |
| 3.1 | 1 | 100% | 0% |
| 20.9 | 1 | 100% | 0% |
| 21 | 1 | 98% | 2% |
| 23 | 1 | 96% | 4% |
| 46 | 1 | 80% | 20% |
| 55 | 1 | 73% | 27% |
| 59 | 1 | 68% | 32% |
| 60.5 | 1 | 55% | 45% |
| 61 | 1 | 5% | 95% |
| 67 | 1 | 100% | 0% |
| 91 | 1 | 100% | 0% |

Solution A: 2% Pierce^TM^ acetonitrile (vol/vol in water; pH 10.0)

Solution B: 90% Pierce^TM^ acetonitrile (vol/vol in water; pH 10.0)

Table S2. Parameters for LC-MS/MS

| Time (min) | Flow rate (nL/min) | Solution A | Solution B |
| --- | --- | --- | --- |
| 0 | 300 | 97% | 3% |
| 5 | 300 | 97% | 3% |
| 6 | 300 | 93% | 7% |
| 37.5 | 300 | 84% | 16% |
| 46 | 300 | 75% | 25% |
| 48 | 300 | 70% | 30% |
| 49 | 300 | 20% | 80% |
| 54 | 300 | 20% | 80% |
| 54.5 | 300 | 97% | 3% |
| 65 | 300 | 97% | 3% |

Solution A: 0.1% Pierce^TM^ formic acid (vol/vol in water)

Solution B: 99.9% Pierce^TM^ acetonitrile (vol/vol in Pierce^TM^ formic acid)

Table S3. Antibodies for WB.

| Name | Brand | Catalog No. | Specificity | Dilution |
| --- | --- | --- | --- | --- |
| DSG1 | Abcam | Ab209490 | Rabbit | 1:1000 |
| SOD2 | Abcam | ab68155 | Rabbit | 1:1000 |

Table S4. DEPs in Pattern 1

| ProbeSetId | Gene_Symbol | Gene_ID | Description | T1 | T2 | T3 |
| --- | --- | --- | --- | --- | --- | --- |
| NP_000578.2 | C7 | 730 | complement component C7 precursor | 1.8383612 | 1.748852 | 2.4372879 |
| NP_001008240.1 | C18orf25 | 147339 | uncharacterized protein C18orf25 isoform b | 1.6828063 | 1.6005042 | 2.8497561 |
| NP_001268374.1 | PKIG | 11142 | cAMP-dependent protein kinase inhibitor gamma | 1.0107877 | 1.0094564 | 6.6957286 |
| NP_001933.2 | DSG1 | 1828 | desmoglein-1 preproprotein | 1.0116209 | 1.0101863 | 6.6866297 |
| NP_001338275.1 | TPCN1 | 53373 | two pore calcium channel protein 1 isoform 3 | 1.0120302 | 1.0105447 | 6.6821676 |
| NP_004416.2 | ECM1 | 1893 | extracellular matrix protein 1 isoform 1 precursor | 1.0143498 | 1.0125762 | 6.6569663 |
| NP_001289599.1 | GSTT2 | 2953 | glutathione S-transferase theta-2 isoform b | 1.0166126 | 1.0145573 | 6.6325292 |
| NP_444295.1 | UBQLN1 | 29979 | ubiquilin-1 isoform 2 | 1.0026928 | 1.0023616 | 6.7938555 |
| NP_001278967.1 | MAN2B2 | 23324 | epididymis-specific alpha-mannosidase isoform 2 precursor | 1.0006934 | 1.0006934 | 6.7923592 |
| NP_067050.1 | OSTC | 58505 | oligosaccharyltransferase complex subunit OSTC isoform 1 | 1.0394255 | 1.0393709 | 3.1890145 |
| NP_115597.3 | CHD6 | 84181 | chromodomain-helicase-DNA-binding protein 6 | 1.007669 | 1.0067238 | 6.7299651 |
| NP_203526.1 | MRPS12 | 6183 | 28S ribosomal protein S12, mitochondrial precursor | 1.0081618 | 1.0071557 | 6.7245367 |
| NP_001184027.1 | PRSS3 | 5646 | trypsin-3 isoform 4 preproprotein | 1.0042912 | 1.0037631 | 6.767363 |
| NP_003722.2 | SSNA1 | 8636 | Sjoegren syndrome nuclear autoantigen 1 | 1.0057172 | 1.0050132 | 6.7515348 |
| NP_444263.1 | MRPS33 | 51650 | 28S ribosomal protein S33, mitochondrial | 1.0556418 | 1.0538261 | 3.1671422 |
| NP_054829.2 | PPP1R8 | 5511 | nuclear inhibitor of protein phosphatase 1 isoform alpha | 1.0518006 | 1.0500209 | 3.1081536 |
| NP_057097.2 | NDUFAF1 | 51103 | complex I intermediate-associated protein 30, mitochondrial precursor | 1.0198236 | 1.0173676 | 6.5980994 |
| NP_001092271.1 | FAM210A | 125228 | protein FAM210A | 1.0526247 | 1.0514212 | 3.1846154 |
| NP_057501.2 | GLRX5 | 51218 | glutaredoxin-related protein 5, mitochondrial precursor | 1.0663366 | 1.065174 | 3.1595424 |
| NP_001171604.1 | SUFU | 51684 | suppressor of fused homolog isoform 2 | 1.1333541 | 1.1228368 | 3.1613002 |
| NP_061897.1 | HEATR5B | 54497 | HEAT repeat-containing protein 5B | 1.1611851 | 1.1498431 | 3.1672471 |
| NP_068839.1 | ITM2B | 9445 | integral membrane protein 2B | 1.0703631 | 1.0602259 | 3.4697689 |
| NP_006440.2 | CDC42EP3 | 10602 | cdc42 effector protein 3 | 1.0872484 | 1.079737 | 3.1319283 |
| NP_000272.1 | PCBD1 | 5092 | pterin-4-alpha-carbinolamine dehydratase isoform 1 | 1.0477007 | 1.0407918 | 3.1037413 |
| NP_001135779.1 | GTF2H1 | 2965 | general transcription factor IIH subunit 1 | 1.0687459 | 1.0615214 | 3.1494186 |
| NP_001007240.1 | EEF1AKNMT | 51603 | eEF1A lysine and N-terminal methyltransferase isoform 3 | 1.0729567 | 1.0696168 | 3.2254807 |
| NP_001337510.1 | TRMT11 | 60487 | tRNA (guanine(10)-N2)-methyltransferase homolog isoform c | 1.0585769 | 1.0529461 | 3.0902364 |
| NP_055299.1 | MOCS3 | 27304 | adenylyltransferase and sulfurtransferase MOCS3 | 1.0515626 | 1.0472022 | 2.9497933 |
| NP_001307127.1 | KRT86 | 3892 | keratin, type II cuticular | 1.5055822 | 1.5797802 | 3.1623826 |
| NP_733821.1 | LMNA | 4000 | lamin isoform A | 1.7439191 | 1.7888598 | 2.4382044 |

Table S5. DEPs in Pattern 2

| ProbeSetId | Gene_Symbol | Gene_ID | Description | T1 | T2 | T3 |
| --- | --- | --- | --- | --- | --- | --- |
| NP_002220.1 | JUNB | 3726 | transcription factor jun-B | 1.6439382 | 2.2640784 | 2.0963871 |
| NP_001892.1 | CCN2 | 1490 | connective tissue growth factor precursor | 1.5892081 | 2.3352358 | 2.0866811 |
| NP_001198.2 | BTF3 | 689 | transcription factor BTF3 isoform B | 1.6639103 | 2.2599405 | 2.0791754 |
| NP_001777.1 | CDK1 | 983 | cyclin-dependent kinase 1 isoform 1 | 1.6350708 | 2.2460327 | 2.1194782 |
| NP_001349969.1 | PBK | 55872 | lymphokine-activated killer T-cell-originated protein kinase isoform 1 | 1.5811444 | 2.2805914 | 2.1457782 |
| NP_001155046.1 | MAFF | 23764 | transcription factor MafF isoform b | 1.6602614 | 2.2230141 | 2.1154797 |
| NP_005333.2 | HMGB3 | 3149 | high mobility group protein B3 isoform a | 1.6315845 | 2.2231909 | 2.1611061 |
| NP_877423.1 | MCM4 | 4173 | DNA replication licensing factor MCM4 | 1.5754989 | 2.2432641 | 2.1487578 |
| NP_005907.3 | MCM7 | 4176 | DNA replication licensing factor MCM7 isoform 1 | 1.600165 | 2.245737 | 2.161045 |

Table S6. DEPs in Pattern 3

| ProbeSetId | Gene_Symbol | Gene_ID | Description | T1 | T2 | T3 |
| --- | --- | --- | --- | --- | --- | --- |
| NP_006050.3 | LAMC3 | 10319 | laminin subunit gamma-3 precursor | 2.377486973 | 1.658595697 | 2.072006731 |
| NP_001153318.1 | MPND | 84954 | MPN domain-containing protein isoform 2 | 2.101616528 | 1.632933493 | 2.327897509 |
| NP_001284706.1 | MCTP1 | 79772 | multiple C2 and transmembrane domain-containing protein 1 isoform 3 | 2.115938362 | 1.640253148 | 2.305167926 |
| NP_002290.2 | LCT | 3938 | lactase-phlorizin hydrolase preproprotein | 2.168971813 | 1.683938978 | 2.20339148 |
| NP_001502.1 | CXCL1 | 2919 | growth-regulated alpha protein precursor [Homo sapiens] | 2.207411061 | 1.69390964 | 2.159652719 |
| NP_588615.2 | ZNF618 | 114991 | zinc finger protein 618 isoform 1 | 2.379254091 | 1.746291654 | 1.972759881 |
| NP_001263343.1 | UNKL | 64718 | putative E3 ubiquitin-protein ligase UNKL isoform 5 | 2.35298096 | 1.749769591 | 1.987196478 |
| NP_000414.2 | KRT2 | 3849 | keratin, type II cytoskeletal 2 epidermal | 2.752423974 | 1.620751183 | 1.884086852 |
| NP_001309749.1 | SOD2 | 6648 | superoxide dismutase [Mn], mitochondrial isoform E | 2.475504135 | 1.725709407 | 1.88334086 |
| NP_003371.2 | VIM | 7431 | vimentin | 2.387607637 | 1.785150749 | 1.915015406 |
| NP_000412.3 | KRT10 | 3858 | keratin, type I cytoskeletal 10 | 2.736112788 | 1.573037403 | 1.940305071 |
| NP_001107579.1 | PDLIM3 | 27295 | PDZ and LIM domain protein 3 isoform b | 2.406338168 | 1.756723832 | 1.943498365 |
| NP_444513.1 | DCD | 117159 | dermcidin isoform 1 preproprotein | 2.392993591 | 1.762364664 | 1.925941169 |
| NP_001293021.1 | EVC | 2121 | ellis-van Creveld syndrome protein isoform 3 | 2.63580365 | 1.667721956 | 1.839962243 |
| NP_066934.1 | RECK | 8434 | reversion-inducing cysteine-rich protein with Kazal motifs isoform 1 precursor | 2.425405613 | 1.791643551 | 1.893397807 |
| NP_064575.1 | OLFML3 | 56944 | olfactomedin-like protein 3 isoform 1 precursor | 2.458279915 | 1.730286411 | 1.852341634 |
| NP_000192.2 | ICAM1 | 3383 | intercellular adhesion molecule 1 precursor | 2.44136188 | 1.782237839 | 1.894586396 |
| NP_005547.3 | KRT7 | 3855 | keratin, type II cytoskeletal 7 | 2.581373732 | 1.75844835 | 1.849527756 |
| NP_000517.3 | KRT14 | 3861 | keratin, type I cytoskeletal 14 | 2.954576575 | 1.59246435 | 1.749578773 |
| NP_000415.2 | KRT5 | 3852 | keratin, type II cytoskeletal 5 | 2.837606588 | 1.632863317 | 1.773057295 |
| NP_473447.1 | OR5AC2 | 81050 | olfactory receptor 5AC2 | 2.894781634 | 1.650952381 | 1.768989318 |
| NP_006112.3 | KRT1 | 3848 | keratin, type II cytoskeletal 1 | 2.969819882 | 1.597832154 | 1.73543288 |
| NP_001264074.1 | ZBTB10 | 65986 | zinc finger and BTB domain-containing protein 10 isoform c | 2.466342626 | 1.796754623 | 1.864453591 |
| NP_005545.1 | KRT6A | 3853 | keratin, type II cytoskeletal 6A | 3.284246433 | 1.567022855 | 1.745463082 |
| NP_001243098.1 | APOM | 55937 | apolipoprotein M isoform 2 | 2.773708774 | 1.68317811 | 1.799445416 |
| NP_057613.4 | ATP8A2 | 51761 | phospholipid-transporting ATPase IB isoform 1 | 2.456058482 | 1.790466294 | 1.877074464 |
| NP_005548.2 | KRT16 | 3868 | keratin, type I cytoskeletal 16 | 3.423830074 | 1.465313649 | 1.723081363 |
| NP_055887.3 | TTLL5 | 23093 | tubulin polyglutamylase TTLL5 | 2.505679595 | 1.769394298 | 1.867024522 |
| NP_003667.1 | DEGS1 | 8560 | sphingolipid delta(4)-desaturase DES1 isoform 1 | 3.29929743 | 1.509207246 | 1.727925517 |
| NP_004325.2 | BST1 | 683 | ADP-ribosyl cyclase/cyclic ADP-ribose hydrolase 2 precursor | 2.493220468 | 1.790235858 | 1.877634916 |
| NP_001264660.1 | AOC3 | 8639 | membrane primary amine oxidase isoform 2 | 2.473720279 | 1.785416524 | 1.870972882 |
| NP_061756.1 | PCDHB13 | 56123 | protocadherin beta-13 precursor | 2.477732249 | 1.774010857 | 1.879300233 |
| NP_001352850.1 | MSL1 | 339287 | male-specific lethal 1 homolog isoform 4 | 2.551734837 | 1.748397466 | 1.859536965 |
| NP_004406.2 | DSP | 1832 | desmoplakin isoform I | 2.490898312 | 1.778220719 | 1.88069404 |

Table S7. DEPs in Pattern 4

| ProbeSetId | Gene_Symbol | Gene_ID | Description | T1 | T2 | T3 |
| --- | --- | --- | --- | --- | --- | --- |
| NP_065084.2 | COL8A1 | 1295 | collagen alpha-1(VIII) chain precursor | 2.2115637 | 2.3240706 | 1.6013584 |
| NP_001337489.1 | HACE1 | 57531 | E3 ubiquitin-protein ligase HACE1 isoform i | 2.2147182 | 2.2817832 | 1.6273471 |
| NP_002657.3 | PLIN1 | 5346 | perilipin-1 | 2.1421248 | 3.0944646 | 1.2558884 |
| NP_006244.2 | PRKAB1 | 5564 | 5'-AMP-activated protein kinase subunit beta-1 | 2.0480496 | 2.4073042 | 1.6505937 |
